# Noninvasive immune-inflammatory profiling by ultrasound radiomics for preoperative prediction of central lymph node metastasis in papillary thyroid carcinoma

**DOI:** 10.3389/fimmu.2026.1912827

**Published:** 2026-09-18

**Authors:** Meng Sun, Jiening Gao, Qian Sui, Meng Li, Yi Wang, Ruoling Han

**Affiliations:** 1 Department of Ultrasound, The Fourth Hospital of Hebei Medical University, Shijiazhuang, China; 2 Department of Clinical Laboratory, The Fourth Hospital of Hebei Medical University, Shijiazhuang, China

**Keywords:** central lymph node metastasis, cross-scale immune corroboration, immune-inflammatory indices, papillary thyroid carcinoma, ultrasound radiomics

## Abstract

**Background:**

Central lymph node metastasis in clinically node-negative papillary thyroid carcinoma is regulated by the tumor immune microenvironment, yet existing studies have addressed molecular, systemic immune, and local imaging signals in isolation without examining their cross-scale associations.

**Methods:**

A retrospective cohort of 1,000 cN0 PTC patients was enrolled. BRAF V600E mutation status, six peripheral blood immune-inflammatory indices, and an ultrasound radiomics deep learning score were integrated into four progressive LightGBM models. Cross-scale Spearman correlations, BRAF-stratified Fisher z-transformation tests, and TCGA-THCA immune deconvolution analysis were performed.

**Results:**

The three-scale integrated model achieved a validation AUC of 0.824, with all DeLong incremental comparisons reaching statistical significance. The DLR-score was significantly correlated with all six immune-inflammatory indices after Bonferroni correction. BRAF-positive subgroup exhibited significantly stronger imaging-immune coupling for NLR and SII, and this enhanced coupling persisted after adjustment for age, tumor size, multifocality, and Hashimoto’s thyroiditis in partial correlation analyses. TCGA tissue immune infiltration patterns were directionally consistent with peripheral observations. Calibration assessment confirmed probability accuracy across the predicted range, and decision curve analysis demonstrated sustained net benefit superiority of the integrated model over single-scale alternatives within the clinically relevant 10% to 50% threshold interval.

**Conclusion:**

Three-scale noninvasive immune signals provide complementary CLNM prediction and exhibit directionally consistent cross-scale statistical associations at the correlation level, suggesting that they may serve as noninvasive surrogates reflecting partially overlapping aspects of the same tumor immune dysregulation process, although confirmation of the underlying biological mechanisms requires prospective studies incorporating direct tissue-level immune profiling.

## 1 Introduction

Papillary thyroid carcinoma (PTC) is the most common type of thyroid malignancy worldwide, characterized by a constantly rising incidence rate, placing it in the topmost causes of cancer amongst women (1). Despite the generally favorable prognosis of PTC, central lymph node metastasis (CLNM) remains a core risk factor for recurrence and reoperation, occurring at a covert rate of 30% to 60% in patients whose preoperative ultrasound assessment indicates clinically node-negative (cN0) status (2). In the body of research obtained through clinical trials and molecular studies, CLNM can be understood not only in terms of spread-related reasons but also from a biological perspective linked to the tumor microenvironment immunology. In this context, the BRAF V600E mutation promotes immune escape by triggering MAPK signaling and is highly correlated with increased risk of lymph node metastasis (3).

Immune dysregulation at a molecular level is similarly expressed by the body’s systemic circulation. Immune-inflammatory markers, such as the neutrophil-to-lymphocyte ratio (NLR) and systemic immune-inflammation index (SII), have been found to be independently correlated with the risk for developing lymph node metastasis in patients suffering from PTC (4). A systematic review with network meta-analysis encompassing multiple inflammatory biomarkers has confirmed the independent predictive value of these indices in PTC prognostic assessment (5). A systematic review and meta-analysis restricted to differentiated thyroid carcinoma pooled 7,599 patients and found no significant association between preoperative NLR, LMR, PLR and disease-free survival, indicating that the prognostic value of these indices across populations remains contested (6). Other types of thyroid cancers have also displayed promising biomarker activity when it comes to these immune-inflammatory markers (7), while new research has highlighted the potential for using pre-operative inflammation biomarkers as predictors of central metastasis in patients diagnosed with cN0 PTC (8). In addition, patients affected by PTC who also suffer from Hashimoto’s thyroiditis show clear differences in metastatic tendencies depending on their specific immune condition (9), thus providing further support to the hypothesis about the involvement of the immune system host condition in PTC metastatic regulation. The collected multi-scale data suggests that mutations caused by BRAF, systemic immune-inflammatory disturbance, and tumor microenvironment remodeling might be different representations of a common immune process at different scales of biology.

Regarding the technical aspect of imaging analysis using ultrasound radiomics, there is a wealth of information suggesting that prediction of metastasis in PTC can be performed with high sensitivity and specificity. This technology was first pioneered based on the validation of radiomic nomograms based on shear wave ultrasound and their additional value in assessing the staging of cervical lymph node staging (10). Afterwards, multi-modal ultrasound imaging fusions were used (11) along with machine learning methods that utilize the SHAP approach to interpret results (12). As for other imaging techniques, there is additional information that can be provided using contrast-enhanced ultrasound radiomics compared to traditional gray scale images (13). Furthermore, development of contrast-enhanced nomograms based on radiomic model has enabled expansion of the technological boundaries (14). The generalizability of radiomic signatures to various patient groups is confirmed by external validation performed in several institutes (15), whereas subgroup analysis provides further proof of the scores’ consistency in heterogeneous populations (16). The structured approach towards the development of prediction scenarios includes integration of clinical-radiomic models (17–19); accurate prognosis within subgroups of cN0 papillary thyroid microcarcinoma (20–22); fusion strategies using multimodal imaging (23, 24); and independent investigation of metastatic spread into lateral neck compartments (25). These all serve as multidimensional clinical evidence that supports the utility of radiomics in preoperative management of PTC.

For the development of technical paradigms, there is an emergence of methods such as end-to-end deep learning (26), super-resolution reconstruction techniques (27), automatic key-frame selection (28), and prediction via AI integrated multi-modal systems (29), thereby shifting the method of prediction from manual feature engineering to representation learning. Predictive targets have also become broader than the lymph node metastasis status prediction and have shifted toward evaluating the risk for recurrence (30) and classifying tumor aggression (31). Meanwhile, combining the two approaches on the dataset level (32), along with their verification through cross-modality using CT radiomics (33, 34), has further widened the scope of usage for imaging biomarkers of PTC. Nevertheless, examining all of these developments through a unified perspective, one can identify an inherent structural problem with the current approach that involves both deep-learning-driven image segmentation (35) and multi-source feature fusion nomograms (36). However, the multi-source properties have consistently been conceived to represent parallel sources, arranged within classifiers (37), where the focus on engineering was on achieving maximum predictability while ignoring whether the signals obtained from different levels show any intrinsic biological connections.

Based on the principles of immunology, correlating quantitative parameters of ultrasound phenotypes and tumor immune microenvironments are biologically plausible. Prior studies based on the foundations of radiomics have shown that quantitative analyses using medical imaging allow for the non-invasive decoding of tumor phenotypes through quantitative features that correspond to tumor heterogeneity (38). The “images are data” principle recognizes that medical imaging not only serves as diagnostic tools but also as high-dimensional data representing the tumor microenvironment (39). In the light of this theory, several studies conducted among different forms of cancers have revealed correlations quantitatively between radiomics and the conditions of the immune microenvironment. For instance, in breast cancer cases, there have been measurable correlations of radiomic features with the distribution patterns of immune cells within the tumors (40). Radiomic signature from MRI has shown potential ability to evaluate the infiltration of tumor-associated macrophages in glioma and predict the efficacy of immunotherapy (41). There have been systematic efforts recently towards establishing the mechanisms behind which AI contributes to precision immunotherapy using the combination of immunogenomics, radiomics, and pathomics (42). The aforementioned cross-cancer data provide theoretical justification for the core hypothesis that ultrasonic radiomics can be used to evaluate the immune-inflammatory profile of PTC. Nevertheless, in the current PTC literature, there is a lack of research efforts that have considered molecular and systemic as well as local noninvasive immune assessment.

Based on the above background information of previous research and the existing academic gap, this study applied a retrospective cohort of 1,000 patients with cN0 PTC in the Fourth Hospital of Hebei Medical University along with a non-invasive tri-scale immune signal cross-corroboration framework. This system aimed to incorporate the BRAF V600E mutation test result, peripheral immune-inflammatory indices (NLR, PLR, LMR, SII, SIRI, and PIV), and an ultrasound radiomics deep learning score (DLR-score), which includes three scores for non-invasive immune-associated indicators, into an CLNM predictive model before surgery. It was also improved to measure the separate contribution of each scale through the progressive model design with four groups, alongside the DeLong incremental method. Furthermore, it was designed to assess the associations among cross-scales using the Spearman correlation analysis of radiomic and immune-inflammatory indicators, BRAF-stratified moderation effect assessment, and validation of tissue-immune infiltrates through TCGA-THCA dataset. As opposed to the existing paradigm that treats predictive performance optimization as the end goal, the key advance of the present study lies in shifting from a predictive focus to a relational one. The research investigates whether directionally consistent statistical associations exist among immune-related signals captured at different biological scales, rather than establishing direct mechanistic causation. Given the retrospective observational design, the cross-scale analyses are positioned as hypothesis-generating evidence intended to motivate subsequent prospective functional validation. The present approach is expected to provide a methodological reference for studying PTC metastasis from an immunological perspective and to offer preliminary multi-scale evidence that may inform individualized preoperative decision-making for cN0 PTC patients.

## 2 Materials and methods

### 2.1 Study population

This was a single-center retrospective observational study approved by the Ethics Committee of the Fourth Hospital of Hebei Medical University (approval number 2023KY078). Since this is a retrospective study, informed consent was waived. The study enrolled patients who underwent surgery between January 2019 and June 2024 and met all of the following criteria: pathologically confirmed PTC, preoperative ultrasound assessment of cN0, thyroidectomy combined with central lymph node dissection, availability of a complete blood routine within one week before surgery, and retrievable preoperative grayscale thyroid ultrasound DICOM images from the PACS system. Exclusion criteria included those patients with other malignancies, neck surgery or radiation therapy before, any infection occurring in the previous two weeks before surgery, immunosuppressant drugs, hematologic disorders, and ultrasound images of inadequate quality.

The number of records to be exported was 1,120 cases, out of which only 1,000 cases qualified based on the eligibility criteria. Based on the chronological order of the surgeries, 731 cases from January 2019 to December 2022 were selected for the training data, whereas 269 cases from January 2023 to June 2024 were deliberately held out as a temporal validation set. Temporal splitting was adopted in preference to random splitting because it more rigorously tests generalizability by exposing the model to patients from a later calendar period who may reflect secular trends in referral patterns, imaging protocols, and surgical practice, thereby mimicking the real-world clinical deployment scenario in which a model trained on historical data is applied to future patients. CLNM was defined as the presence of at least one positive lymph node in the central compartment (level VI) on postoperative paraffin-section pathology. Since 2020, the institute has regularly been performing BRAF V600E screening for PTC samples via real-time fluorescence-based PCR, yielding an 85.2% BRAF test coverage rate in the cohort (852 of 1,000 patients). The missingness of BRAF results was predominantly driven by the institutional adoption timeline of routine molecular testing rather than by patient-level clinical characteristics, constituting a mechanism-based missing pattern tied to calendar period. Given that BRAF V600E is a somatic tumor mutation whose status cannot be reliably predicted from routine clinical or hematological variables, standard multiple imputation was considered methodologically inappropriate for this context, as imputing a binary molecular marker from clinically unrelated predictors would introduce uncertain assumptions and potentially distort the very associations under investigation. For the predictive modeling framework, a missing indicator approach was adopted whereby patients with unavailable BRAF results were encoded as BRAF-negative in the mutation status variable together with a companion binary missing indicator set to 1, enabling the model to distinguish BRAF-negative patients from those with unknown status and to retain the full 1,000-patient cohort for training and validation without discarding cases or imputing unverifiable mutation status. For the BRAF-stratified cross-scale correlation analyses, which required definitive BRAF classification, a complete-case analytical strategy was adopted within the 852-patient sub-cohort with available test results (BRAF-positive n = 505, BRAF-negative n = 347). To further evaluate the contribution of BRAF information, a sensitivity analysis was performed using Model D reconstructed without either BRAF status or the missing indicator as input variables. The overall study design and analytical framework are illustrated in **Figure 1**.

**Figure 1:**
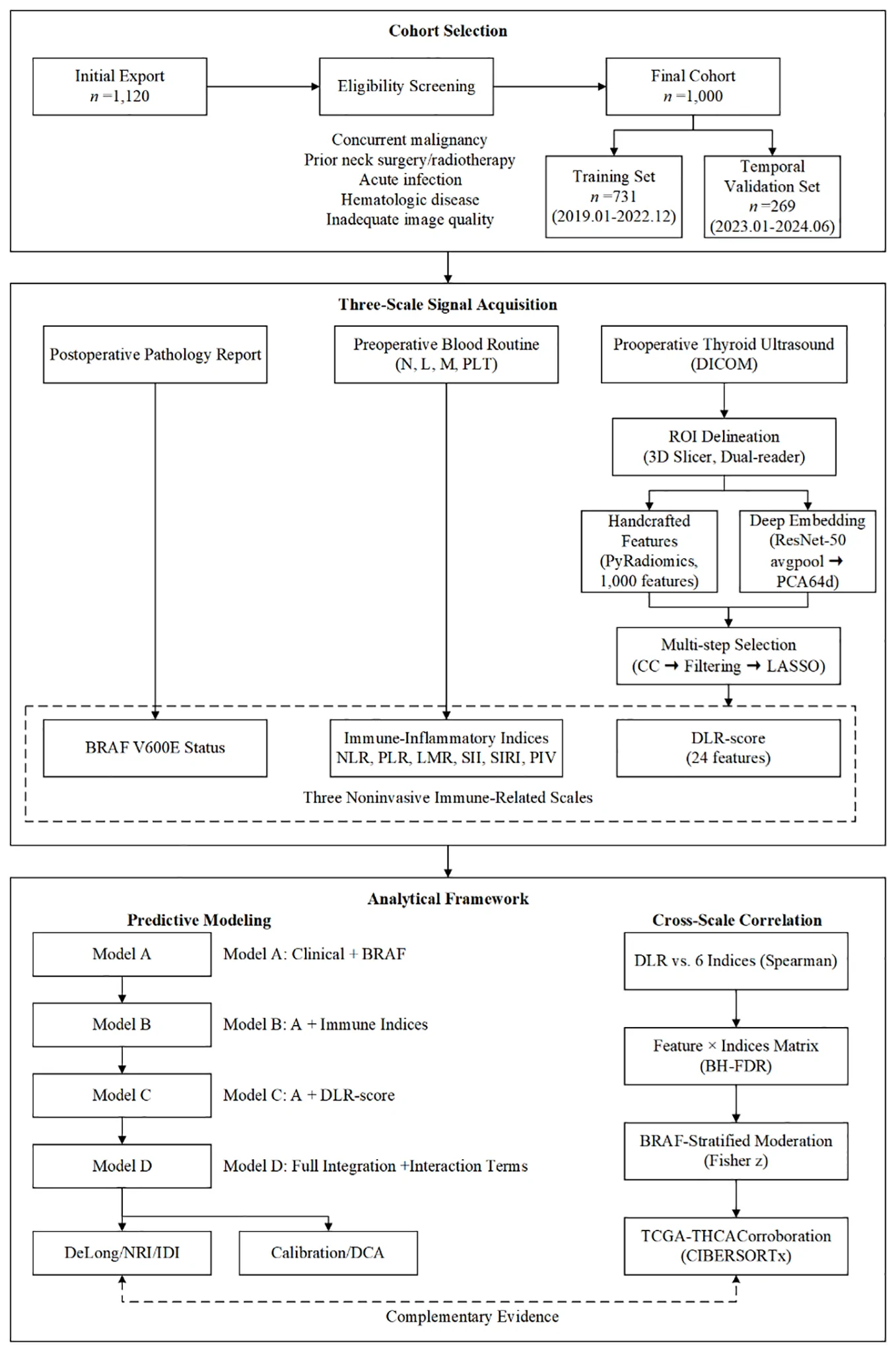
Study design and analytical framework.

### 2.2 Clinical and immune-inflammatory variables

The clinical variables were collected on the basis of the criteria set forth in the management guidelines provided by the American Thyroid Association in 2015 (43). These include age, gender, maximum tumor diameter (ultrasound-measured, mm), multifocality, capsular invasion, concurrent Hashimoto’s thyroiditis (pathologically determined), and ACR TI-RADS classification. BRAF V600E mutation status, acting as the molecular-level marker, was assessed using postoperative pathologic findings. Peripheral blood immune-inflammatory indices were calculated from routine complete blood counts (neutrophil count N, lymphocyte count L, monocyte count M, and platelet count PLT, all in ×10^9^/L) collected under standardized fasting morning conditions between 6:00 and 8:00 AM within seven days prior to surgery, thereby minimizing circadian and postprandial fluctuations in leukocyte subpopulations. Patients receiving systemic corticosteroids or immunosuppressive agents at the time of sampling were excluded per the eligibility criteria. Although overt infections within two weeks before surgery were excluded, subclinical chronic inflammatory conditions and baseline metabolic profiles such as diabetes mellitus, obesity, and dyslipidemia were not systematically recorded in the retrospective dataset and could not be adjusted for, representing a residual confounding source acknowledged in the limitations. Following the original definition of the systemic immune-inflammation index (44), six indices were computed as shown in Equations 1, 2:

(1) $$\begin{array}{ccc} NLR=N/L, & PLR=PLT/L, & LMR=L/M \end{array}$$

(2) $$\begin{array}{ccc} SII=PLT\times N/L, & SIRI=N\times M/L, & PIV=PLT\times N\times M/L \end{array}$$

These indices were categorized into two tiers according to their compositional complexity and immunological interpretation. The three binary ratios (NLR, PLR, and LMR) each capture the balance between two circulating cell lineages and primarily reflect the equilibrium between innate pro-inflammatory effectors and adaptive immune surveillance at the systemic level. The three composite indices (SII, SIRI, and PIV) integrate three to four cell lineages into a single metric, providing a more comprehensive representation of the overall systemic immune-inflammatory burden. It should be noted that all six indices are derived from peripheral blood absolute counts of circulating leukocyte subsets and platelets, and as such they serve as surrogate markers of systemic broad-spectrum inflammation rather than direct indicators of local adaptive immunity, antigen-specific T cell responses, or tumor immune evasion mechanisms within the thyroid microenvironment. The rationale for their inclusion in the present framework is not to claim equivalence with tissue-level immune profiling, but to test the hypothesis that systemic immune-inflammatory perturbations captured by routine hematological parameters exhibit directionally consistent associations with local imaging heterogeneity features that may co-vary with the tumor microenvironment. All six indices were included in the statistical analyses as continuous measures, and their cutoff points were additionally computed by X-tile analysis and presented for descriptive purposes.

### 2.3 Ultrasound radiomics pipeline

Considering the methodological framework of radiomics that acts as the connection between medical imaging and personalized medicine (45), a two-path pipeline was designed for simultaneous extraction of handcrafted radiomics and deep learning embedded features. Preoperative grayscale DICOM ultrasound images of thyroid were selected from the PACS archive database, and the maximum cross-sectional area of the tumor was determined. Two sonographers, each with more than five years of experience in thyroid ultrasound, independently delineated the full tumor region of interest (ROI) using 3D Slicer (v5.x). Intraclass correlation coefficients (ICC) were computed on a random subset of 30 cases, and only features with ICC ≥ 0.75 were retained for subsequent analysis.

Handcrafted radiomic features were extracted using PyRadiomics (v3.1.0, IBSI-compliant). Images acquired from five ultrasound device models (Aplio i800, EPIQ 7, LOGIQ E10, LOGIQ E9, and Resona 7) were preprocessed with 0.5 mm × 0.5 mm isotropic resampling and μ ± 3σ gray-level normalization (bin width = 25) to mitigate inter-device gray-level distribution differences before feature extraction. Seven feature classes (first-order, shape2D, GLCM, GLRLM, GLSZM, NGTDM, and GLDM) were extracted at three levels: original images, LoG-filtered images (σ = 1, 2, 3 mm), and first-level wavelet decomposition (8 subbands), yielding a total of 1,000 handcrafted radiomic features. Deep embedding features were extracted from the average pooling layer of an ImageNet-pretrained ResNet-50, producing 2,048-dimensional feature vectors that were subsequently reduced to 64 dimensions via PCA retaining 95% of the cumulative variance.

The combined pool of 1,064 handcrafted and deep PCA features was subjected to a multi-step selection pipeline conducted strictly within the training cohort to prevent information leakage. All preprocessing steps including isotropic resampling, gray-level normalization, and PCA dimensionality reduction were fitted exclusively on training set parameters and subsequently applied to the temporal validation set without refitting. Within the training cohort, 954 features (89.7%) passed the ICC ≥ 0.75 stability screening, after which near-zero variance filtering and redundancy removal based on Pearson correlation coefficients exceeding 0.90 in absolute value were performed. The remaining features were jointly screened via LASSO-logistic regression (10-fold cross-validation within the training set, $\lambda =\lambda_{1_{se}}$). The one-standard-error penalty was chosen to favor a parsimonious yet informative feature subset, balancing model complexity against predictive performance by selecting the most regularized model whose cross-validated deviance remained within one standard error of the minimum. A final set of 24 features was retained, a number consistent with the events-per-predictor guideline of approximately 10 to 20 events per variable recommended for stable logistic regression estimation, given that the training cohort contained 300 CLNM-positive events yielding approximately 12.5 events per retained predictor. These 24 features were combined into a unified deep learning radiomic score (DLR-score) through weighted linear combination using the LASSO regression coefficients. The DLR-score formula derived from the training cohort was then applied to the temporal validation cohort as a locked model without any parameter re-estimation, and all LightGBM hyperparameter optimization via Optuna was likewise restricted to the training cohort. The complete dual-pathway feature extraction and selection pipeline is illustrated in **Figure 2**.

**Figure 2:**
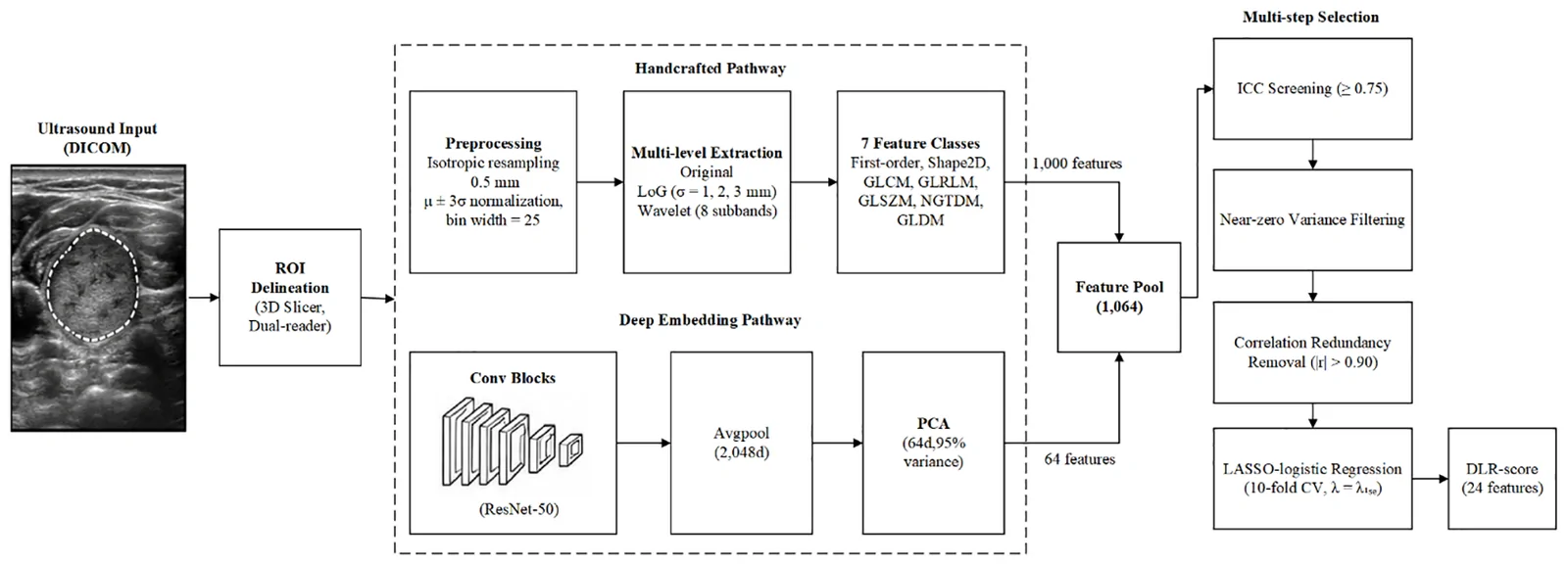
Radiomics feature extraction and selection pipeline.

**Figure 2** illustrates the complete technical pipeline from ultrasound DICOM image input through ROI delineation into dual-pathway parallel feature extraction, where the handcrafted pathway extracts seven classes of textural features across three filtering levels yielding 1,000 dimensions and the deep embedding pathway produces 64 dimensions via ResNet-50 and PCA reduction. The two pathways converge into a 1,064-dimensional feature pool that undergoes stepwise compression through four successive stages of ICC stability screening, variance filtering, correlation redundancy removal, and LASSO regression, ultimately converging to 24 selected features combined into a unified DLR-score through coefficient weighting.

### 2.4 Model construction and incremental validation

To quantify the independent contribution of each immune-related scale, four progressive models were designed. Model A incorporated clinical variables and BRAF as the molecular-scale baseline. Model B added the six immune-inflammatory indices to Model A to introduce the systemic immune scale. Model C added the DLR-score to Model A to introduce the local imaging scale. In Model D, all three models were incorporated, as well as interactions of the DLR-score with SII, NLR, and LMR, and BRAF with SII and PIV, for modeling the nonlinear interactions across scales. All four models incorporated BRAF V600E status alongside the binary BRAF missing indicator variable described in Section 2.1, enabling the use of the full 1,000-patient cohort for model training (n = 731) and temporal validation (n = 269) without excluding patients with unavailable BRAF results. A sensitivity analysis was performed by reconstructing Model D after removing both BRAF V600E status and the companion missing indicator from the input feature set while retaining all remaining variables, and applying this reduced model to the same full 1,000-patient cohort (training n = 731, temporal validation n = 269) to evaluate whether the core predictive performance of the integrated framework was dependent on the availability of molecular-level BRAF information. The BRAF-stratified cross-scale correlation analyses described in Section 2.5, which required definitive BRAF classification, were performed within the 852-patient sub-cohort with available test results (BRAF-positive n = 505, BRAF-negative n = 347). All four algorithms utilized the identical LightGBM classifier (Optuna hyperparameter optimization, 5-fold stratified cross-validation), differing only in terms of the input features, thus ensuring that any performance discrepancy was due to the scaling difference alone.

Discriminative capability was assessed primarily through AUC, with further support coming from accuracy, sensitivity, specificity, and F1 scores. Three important incremental comparisons were conducted using the validation cohort by DeLong tests, whereby B versus A measured the benefit of the increment of the systemic immune score, D versus C assessed the gain due to including systemic immune indices in addition to imaging and molecular markers, and D versus B measured the gain from imaging markers. Additional calculations were conducted using continuous net reclassification improvement (NRI) and integrated discrimination improvement (IDI) to measure the practical benefits of the model on the decision-making process. SHAP was applied to evaluate global features importance as well as predictions for each individual case. The performance of calibration was analyzed via calibration plots in combination with the Hosmer-Lemeshow test, whereas the usefulness of the prediction model from a clinical perspective was examined by decision curve analysis (DCA). To ensure the robustness of the classification model selection, LR, RF, and XGBoost were used as complementary models based on the same input features, while a sensitivity analysis was carried out for Model D without BRAF on the whole dataset. Further subgroup stratification was also performed in the validation cohort according to tumor size (PTMC versus non-PTMC), presence or absence of Hashimoto’s thyroiditis, BRAF mutation, and sonography equipment used to explore the predictive performance stability of Model D in various clinical settings and imaging instruments.

### 2.5 Cross-scale immune correlation analysis

Regarding the overall association level, the DLR score, acting as an integrated quantitative measure of the heterogeneity of the local lesion on radiological images, was associated with each of the six systemic immune-inflammatory indices (NLR, PLR, LMR, SII, SIRI, and PIV) using Spearman rank correlation tests, with the significance threshold set at α = 0.0083 after Bonferroni correction for six comparisons. This analysis aimed to determine whether imaging textural features and peripheral immune-inflammatory status exhibited a directionally consistent association. At the individual feature level, Spearman correlations were computed between six immune-inflammatory indices and the 10 highest-weighted radiomic features among the 24 features retained by LASSO, yielding a 10 × 6 correlation matrix. Multiple comparisons were adjusted across the 60 feature-index correlation tests. This feature-level analysis was designed to investigate whether the observed DLR-score-level associations were driven by a small number of isolated features or reflected a broader correlation pattern within the selected radiomic feature set.

At the BRAF-stratified moderator level, the BRAF sub-cohort was further separated into mutation-positive and wild type groups, and the Spearman correlation coefficient between the DLR score and NLR, SII, and LMR was calculated separately for each group. The statistical significance of the difference between the two sets of correlation coefficients was tested using Fisher z-transformation. This stratified analysis was based on the biological premise that if BRAF V600E serves as an upstream driver of immune evasion in PTC, its positive status should strengthen the coupling between imaging textural features and systemic immune-inflammatory signals, manifesting as stronger DLR-score-to-immune-index correlations in the BRAF-positive group.

To address the possibility that the observed differences in correlation strength between BRAF subgroups were attributable to confounding by clinical covariates known to simultaneously influence peripheral inflammatory levels and imaging heterogeneity, a supplementary partial correlation analysis was performed. Partial Spearman rank correlations between DLR-score and NLR, SII, and LMR were computed separately within BRAF-positive and BRAF-negative subgroups after adjusting for age, maximum tumor diameter, multifocality, and Hashimoto’s thyroiditis status. The adjusted correlation coefficients were compared between the two BRAF subgroups using Fisher z-transformation applied to the partial correlations, with degrees of freedom reduced by the number of covariates controlled.

At the tissue-level corroboration level, RNA-seq gene expression profiles for the TCGA-THCA project were retrieved from the Genomic Data Commons Data Portal (GDC, https://portal.gdc.cancer.gov/, Data Release 45.0, accessed January 15, 2026). HTSeq-FPKM values were downloaded and converted to transcripts per million (TPM) followed by transformation for normalization. Cases were filtered to retain only those with a histological diagnosis of papillary thyroid carcinoma and available somatic BRAF V600E mutation annotation derived from the TCGA PanCancer Atlas mutation dataset, yielding a final analytic cohort of 507 PTC cases (BRAF-positive n = 280, BRAF-negative n = 227). Estimated proportions of 22 immune cell types were obtained by submitting the normalized expression matrix to the CIBERSORTx web tool (https://cibersortx.stanford.edu/, accessed January 2026) using the LM22 signature matrix with 1,000 permutations for statistical significance testing, applying B-mode batch correction, and filtering results at a deconvolution *P* < 0.05 to retain only samples with reliable estimated fractions. Immune infiltration profiles were compared between BRAF mutation-positive and wild-type groups, with particular attention to neutrophils, CD8^+^ T cells, and M2 macrophages as the three key cell types corresponding to the directional changes in peripheral indices. The directional changes in peripheral immune-inflammatory indices were then assessed for consistency with the TCGA tissue infiltration patterns. This analysis was positioned as tissue-level indirect corroboration from an independent public dataset, providing immunological plausibility for the hypothesis that peripheral blood immune-inflammatory indices may partially reflect the tumor microenvironment immune status, rather than serving as independent external validation of the imaging-based prediction model.

### 2.6 Statistical analysis

Statistical analyses were performed using Python 3.10 (scikit-learn 1.3, lightgbm 4.x, shap 0.42, pyradiomics 3.1.0) and R 4.3 (pROC, rms, dcurves). Normality of continuous variables was assessed using the Shapiro-Wilk test, with normally distributed variables described as mean ± standard deviation and compared using independent-samples t-tests, and skewed variables described as median (interquartile range) and compared using Mann-Whitney U tests. Categorical variables were described as frequencies (percentages) and compared using chi-square tests or Fisher exact tests. Multiple comparison correction employed the Bonferroni method for the overall DLR-score-to-immune-index associations and the Benjamini-Hochberg false discovery rate method for the feature-level correlation matrix. All tests were two-sided, and P < 0.05 was considered statistically significant.

## 3 Results

### 3.1 Cohort characteristics

A total of 1,000 cN0 PTC patients were included after eligibility screening from 1,120 initially exported cases, with 411 (41.1%) classified as CLNM-positive. No statistically significant differences in baseline characteristics were observed between the training set (n = 731) and the temporal validation set (n = 269), confirming that the chronological split did not introduce systematic distributional drift. Since the main premise of the current study is based on the idea that molecular mutation, immunological imbalance, and heterogeneous imaging might indicate one shared mechanism of immune dysregulation, it was critical to find out whether there were any baseline differences between CLNM-positive and CLNM-negative patients regarding these parameters before building the predictive model. This was conducted to determine whether each scale carried sufficient discriminative information, as presented in **Table 1**.

**Table 1:** Baseline characteristics of the study cohorts.

Variable	CLNM− (n = 589)	CLNM+ (n = 411)	P value
Clinicopathological characteristics			
Age, years, mean ± SD	44.3 ± 12.0	41.8 ± 12.1	< 0.001
Female sex, n (%)	467 (79.3)	301 (73.2)	0.031
Tumor size, mm, median (IQR)	10.8 (6.8–14.6)	12.4 (9.3–16.2)	< 0.001
Multifocality, n (%)	142 (24.1)	145 (35.3)	< 0.001
Capsular invasion, n (%)	130 (22.1)	138 (33.6)	< 0.001
Hashimoto’s thyroiditis, n (%)	148 (25.1)	79 (19.2)	0.034
ACR TI-RADS, n (%)			0.015
TR3	116 (19.7)	69 (16.8)	
TR4	379 (64.3)	247 (60.1)	
TR5	94 (16.0)	95 (23.1)	
Molecular variable			
BRAF V600E positive, n/known (%)	274/507 (54.0)	231/345 (67.0)	< 0.001
Peripheral immune-inflammatory indices, median (IQR)			
NLR	1.92 (1.62–2.34)	2.22 (1.84–2.71)	< 0.001
PLR	128.74 (107.28–150.70)	144.94 (123.18–175.15)	< 0.001
LMR	4.35 (3.57–5.22)	3.70 (3.12–4.54)	< 0.001
SII	447.40 (350.01–571.89)	549.69 (430.64–691.36)	< 0.001
SIRI	0.81 (0.63–1.04)	1.02 (0.77–1.26)	< 0.001
PIV	184.45 (140.51–257.76)	254.22 (178.82–331.18)	< 0.001

Continuous variables: mean ± SD or median (IQR). Categorical variables: n (%). BRAF positivity among patients with available results (coverage 85.2%). NLR, neutrophil-to-lymphocyte ratio; PLR, platelet-to-lymphocyte ratio; LMR, lymphocyte-to-monocyte ratio; SII, systemic immune-inflammation index; SIRI, systemic inflammation response index; PIV, pan-immune-inflammation value.

**Table 1** shows that the median NLR and SII in the CLNM-positive group were 2.22 and 549.69, significantly higher than 1.92 and 447.40 in the CLNM-negative group, while LMR decreased from 4.35 to 3.70, indicating a systemic immune-inflammatory shift toward a pro-tumor direction. This pattern was directionally consistent with the increase in BRAF positivity rate from 54.0% to 67.0%, suggesting that molecular driver mutation and systemic immune imbalance may synergistically contribute to the development of CLNM.

### 3.2 Feature selection and model performance

Following the completion of DLR-score construction through the dual-pathway feature extraction and multi-step selection pipeline, the central question was how much independent discriminative increment each of the three noninvasive immune scales contributed to CLNM prediction, and whether full integration outperformed any single- or dual-scale combination. To address this question, four progressive models were designed to differ only in the input variables while maintaining identical classifiers and hyperparameter search strategies, thereby strictly attributing performance differences to scale-specific increments rather than algorithmic variation. The validation set discrimination performance is presented in **Figure 3**.

**Figure 3:**
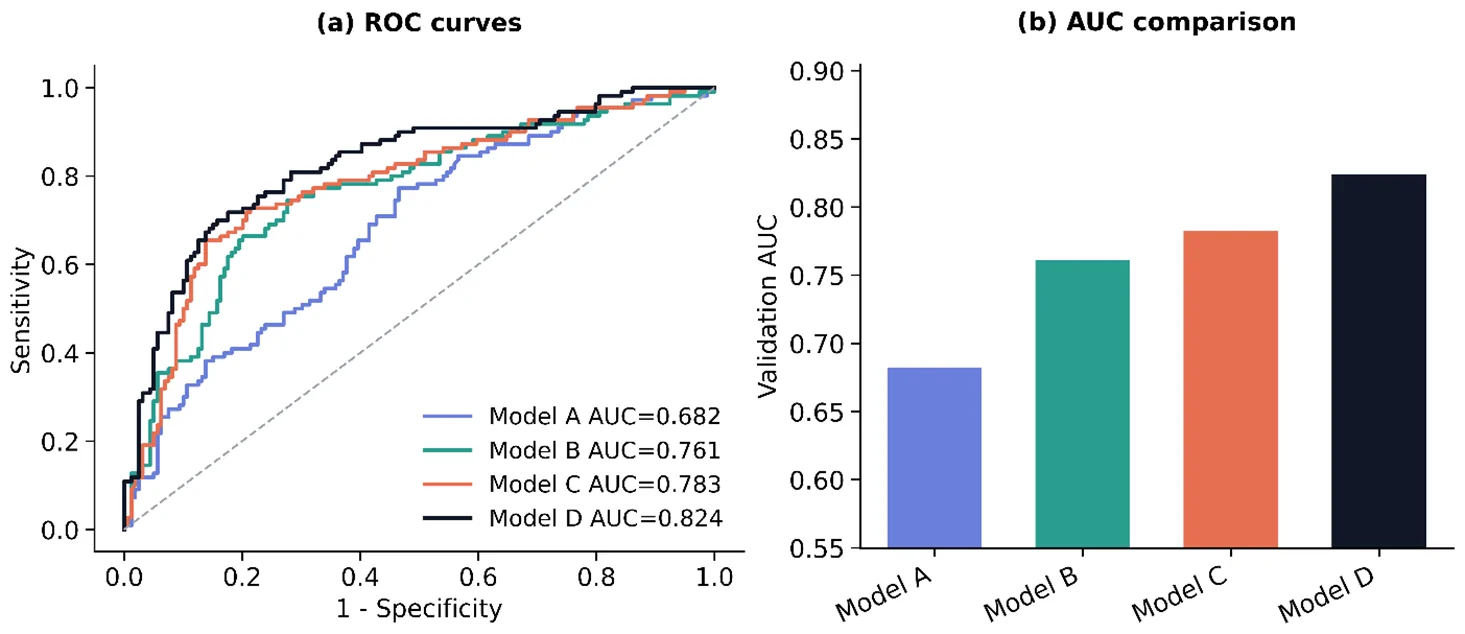
Discrimination performance of the four progressive models.

As shown in (**Figure 3a**), the ROC curve for the models is arranged in a stratified fashion following the sequence of scales. For example, the ROC curve of Model A approaches the diagonal, while that of Model B and C switches position within the middle, with Model D occupying the position nearest to the top left of the graph irrespective of the range of specificity levels. This indicates that the three-level combination model maintains an optimal balance between sensitivity and specificity. The comparative bar graph shown in (**Figure 3b**) better illustrates the successive growth in AUC for models ranging from A to D, where every subsequent immune scale adds value and does not result in diminishing marginal utility. This consistent incremental trend shows that mutation at the molecular level, inflammation of the immune system, and image heterogeneity at the regional level each make an essential, unique contribution towards predicting CLNM, thus confirming the key proposition that information obtained at the three levels is complementary rather than redundant.

The significance of the improvements that occurred in the discrimination performance, as well as whether they provided any meaningful improvement at the decision-making level, needed to be ascertained through quantitative measures of increment and reclassification improvement. Additionally, the sensitivity of the core conclusions to classifier selection and BRAF information availability directly affects the generalizability of the findings, as presented in **Table 2**.

**Table 2:** Diagnostic metrics and incremental validation of the four progressive models.

Model	AUC (95% CI)	Sensitivity (95% CI)	Specificity (95% CI)	Accuracy (95% CI)	F1 (95% CI)
Model A	0.682 (0.618–0.746)	0.509 (0.417–0.607)	0.698 (0.630–0.772)	0.621 (0.561–0.680)	0.523 (0.442–0.603)
Model B	0.761 (0.702–0.821)	0.636 (0.539–0.725)	0.811 (0.751–0.869)	0.740 (0.688–0.792)	0.667 (0.588–0.735)
Model C	0.783 (0.725–0.841)	0.727 (0.644–0.807)	0.786 (0.722–0.850)	0.762 (0.714–0.810)	0.714 (0.644–0.776)
Model D	0.824 (0.772–0.876)	0.736 (0.655–0.817)	0.774 (0.706–0.838)	0.758 (0.706–0.807)	0.714 (0.642–0.776)
Model D w/o BRAF	0.793 (0.736–0.849)	0.764 (0.678–0.840)	0.780 (0.712–0.844)	0.773 (0.717–0.825)	0.734 (0.663–0.797)
LR	0.799 (0.744–0.854)	—	—	—	—
RF	0.801 (0.747–0.855)	—	—	—	—
XGBoost	0.779 (0.722–0.836)	—	—	—	—

All metrics on temporal validation set (n = 269). 95% CI for AUC computed via DeLong method; 95% CI for sensitivity, specificity, accuracy, and F1 computed via 2,000-iteration bootstrap resampling. DeLong: B vs A ΔAUC = 0.079 P = 0.006, D vs C ΔAUC = 0.042 P = 0.008, D vs B ΔAUC = 0.063 P = 0.016. D vs A NRI = 0.661 (95% CI 0.444–0.869), IDI = 0.167 (95% CI 0.121–0.213).

**Table 2** demonstrates that all three DeLong incremental comparisons reached statistical significance, with the addition of immune-inflammatory indices yielding the largest single-step increment of ΔAUC = 0.079 and P = 0.006, suggesting that the systemic immune scale carries independent predictive information not captured by clinical and molecular variables and provides the strongest complementary effect to the baseline model. The ΔAUC of 0.042 with P = 0.008 for D versus C indicates that immune indices contribute an irreplaceable increment even when imaging radiomics has already been incorporated, corroborating at the prediction level the complementarity rather than redundancy of information encoded by systemic immune-inflammatory signals and local imaging textural features. The primary advantage of Model D over single- and dual-scale models was reflected in its superior AUC of 0.824 and improved sensitivity of 0.736, whereas its accuracy of 0.758 was marginally lower than that of Model C at 0.762, and its F1 score of 0.714 was identical to Model C, indicating that the three-scale integration primarily enhanced discriminative capacity and detection sensitivity rather than uniformly outperforming across all classification metrics. The NRI of 0.661 (95% CI 0.444–0.869) and IDI of 0.167 (95% CI 0.121–0.213) for Model D relative to Model A indicate that three-scale integration not only improves discriminatory power but also reclassifies a substantial proportion of patients into more appropriate risk categories based on immune and imaging signals, with both reclassification metrics retaining statistical significance as evidenced by confidence intervals excluding zero. Regarding robustness, the sensitivity analysis of Model D reconstructed without BRAF input on the full 1,000-patient cohort achieved an AUC of 0.793 (95% CI 0.736–0.849) with sensitivity of 0.764 (95% CI 0.678–0.840), specificity of 0.780 (95% CI 0.712–0.844), accuracy of 0.773 (95% CI 0.717–0.825), and F1 of 0.734 (95% CI 0.663–0.797). Compared with the full Model D, the AUC decreased by 0.031 while accuracy improved marginally from 0.758 to 0.773 and F1 improved from 0.714 to 0.734, indicating that removal of BRAF information attenuated discriminative capacity modestly but did not compromise overall classification performance, thereby confirming that the core predictive framework is not dependent on BRAF data availability. LR, RF, and XGBoost using the same Model D input features provided AUC values in the range of 0.779 to 0.801, all directionally consistent with the LightGBM results, confirming that the core findings are likewise not contingent on classifier choice.

The discriminatory stability of a particular model among diverse patient groups and under different equipment acquisition circumstances is what decides its applicability in a clinical setting. The present cohort encompassed five ultrasound device models (Aplio i800, EPIQ 7, LOGIQ E10, LOGIQ E9, and Resona 7) and included diverse clinical phenotypes spanning PTMC and non-PTMC as well as Hashimoto and non-Hashimoto presentations, providing natural stratification conditions for evaluating the cross-scenario stability of Model D, as presented in **Figure 4**.

**Figure 4:**
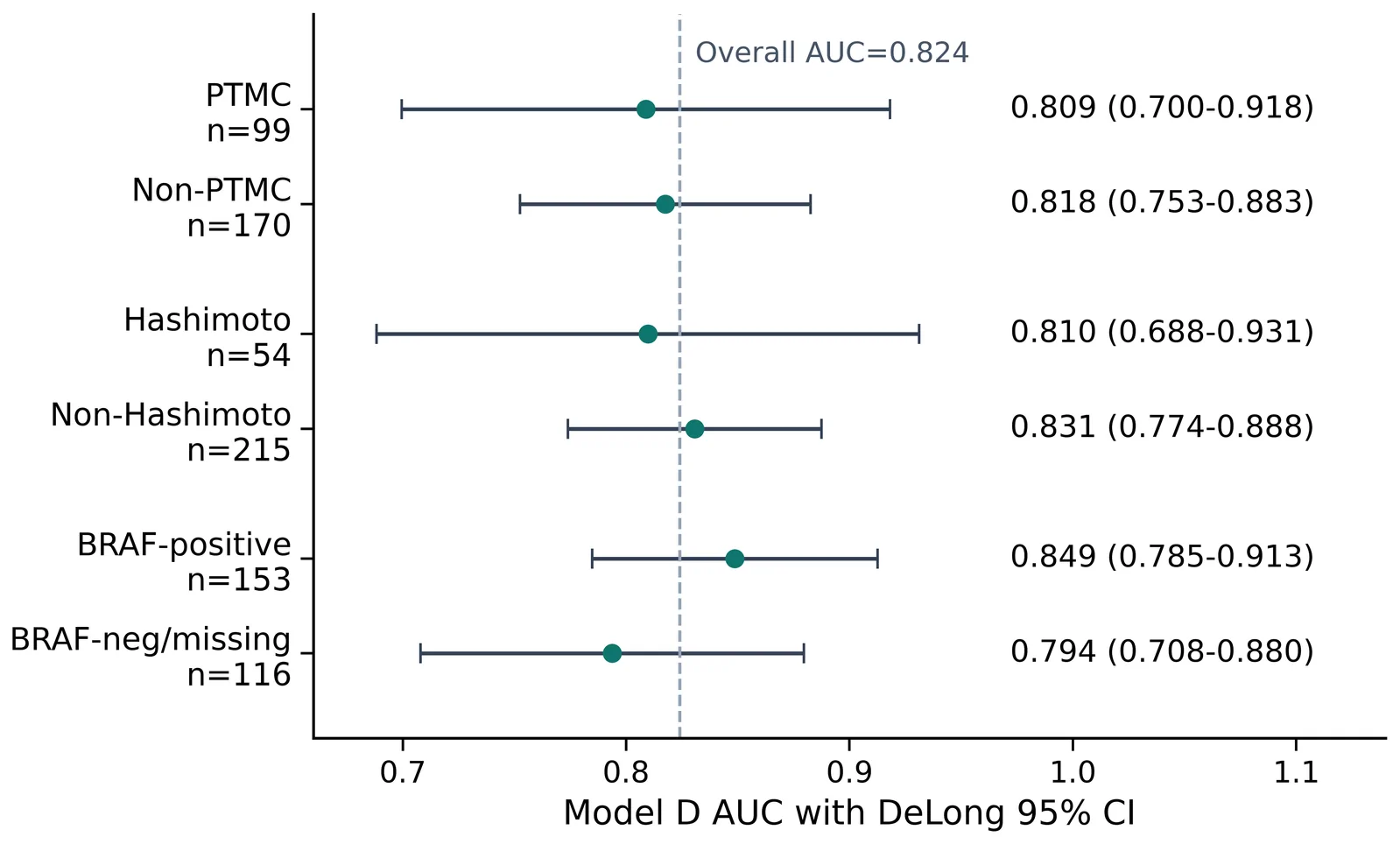
Subgroup performance of the integrated model. 95% CI computed via DeLong method. Dashed vertical line indicates overall Model D AUC of 0.824.

**Figure 4** shows that the AUC of Model D remained above 0.79 across all six clinical subgroups in the temporal validation set, ranging from 0.794 with 95% CI of 0.708 to 0.880 in the BRAF-negative/missing subgroup to 0.849 with 95% CI of 0.785 to 0.913 in the BRAF-positive subgroup, with overlapping confidence intervals across all pairwise comparisons indicating that the discriminative performance of the three-scale integrated model is not substantially affected by tumor size, autoimmune status, or molecular subtype. The BRAF-positive subgroup exhibited the highest point estimate while the BRAF-negative/missing subgroup showed the lowest, a difference that may reflect the enhanced multi-scale signal coupling provided by BRAF-associated immune microenvironment remodeling, enabling more effective utilization of complementary information across the three scales in this subpopulation.

### 3.3 Model interpretability

For the clinical validity of a prediction model, it is not only necessary that it possess good discriminative ability but also that it agrees with the underlying biology and imaging in its reasoning process. If the three-scale integrative model does represent immune-relevant predictive information, the interaction effects between imaging and immune factors should take precedence in the global feature importance hierarchy, and explanations at the individual case level would need to reveal the effect of the various scales on prediction, as depicted in **Figure 5**.

**Figure 5:**
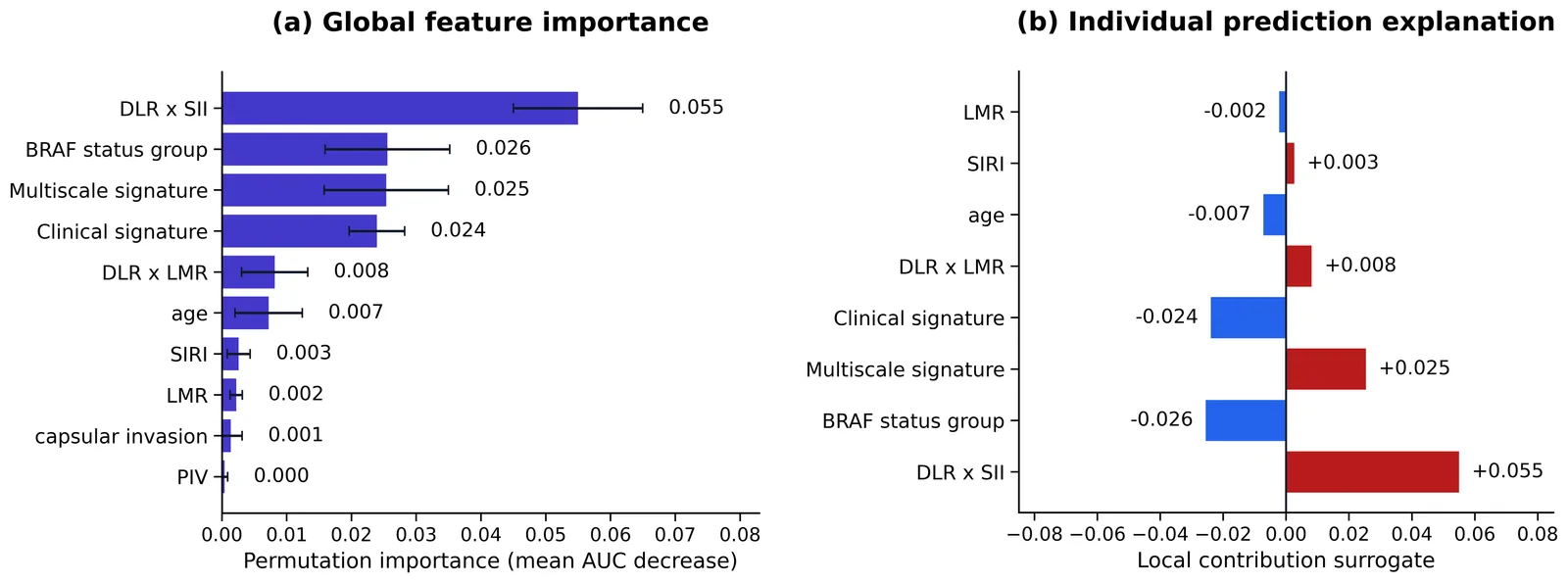
Global feature importance and individual prediction explanation.

Based on the global importance ranking illustrated in (**Figure 5a**), it is observed that the interaction term in DLR and SII far exceeds all other features in significance and holds the top ranking spot. The BRAF feature set, multi-scale signature, and clinical signature follow suit while the independent significance of individual immune and clinical features remains relatively limited. The prediction made by the model seems to be mainly based on interaction terms across scales rather than any single variable. The individual explanation in (**Figure 5b**) further reveals that at the level of specific cases, signals from different scales can exert synergistic or antagonistic effects on the prediction direction, where the DLR-SII interaction term and multiscale signature strongly push toward a positive prediction while the clinical signature and BRAF generate opposing forces. This antagonistic pattern reflects the dynamic interplay mechanism among three-scale signals in individual decision-making and also explains why the fully integrated model can identify heterogeneous cases more accurately than single-scale models.

### 3.4 Cross-scale immune corroboration

The SHAP analysis revealed the dominant role of imaging-immune interaction terms in prediction, yet feature importance rankings within the model cannot directly establish that imaging textural features and peripheral immune-inflammatory status share an intrinsic biological association independent of the prediction task. To systematically examine this association outside the model framework, Spearman rank correlation analyses were performed between DLR-score and each of the six systemic immune-inflammatory indices. Furthermore, if BRAF V600E as an upstream driver of immune evasion in PTC indeed strengthens the coupling between imaging and immune signals, a stronger DLR-score-to-immune-index association should be observed in the BRAF-positive subgroup, as presented in **Figure 6**.

**Figure 6:**
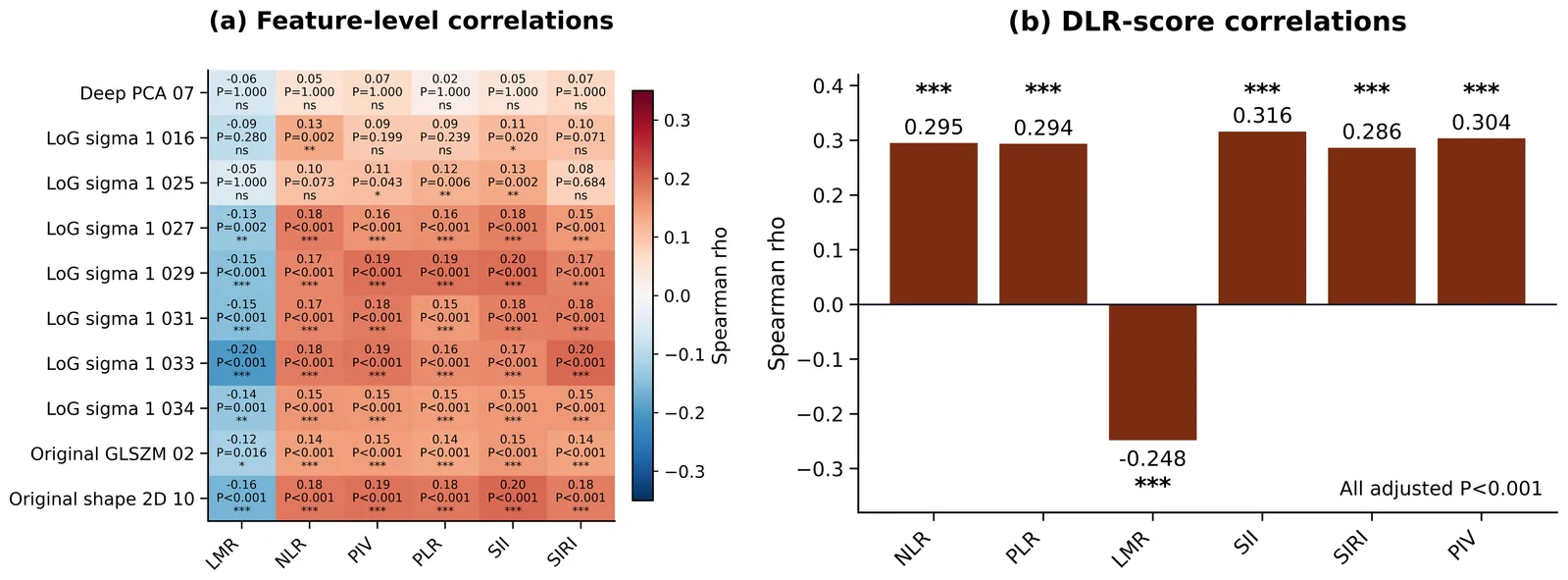
Cross-scale correlation between radiomic features and immune-inflammatory indices. *P < 0.05, **P < 0.01, ***P < 0.001; ns, not significant. P values in **(a)** corrected by Benjamini-Hochberg FDR; P values in **(b)** corrected by Bonferroni method.

The feature-level correlation matrix in (**Figure 6a**) demonstrates that all ten selected radiomic features are positively correlated with NLR, SII, PIV, PLR, and SIRI while negatively correlated with LMR. This highly systematic directional consistency indicates that the imaging-immune association is an intrinsic property of the radiomic feature set as a whole rather than being driven by a few outlier features, with LoG-filtered textural features exhibiting the most prominent association strength, suggesting that tumor textural heterogeneity is most closely linked to immune imbalance. (**Figure 6b**) converges this feature-level pattern to the overall DLR-score level, where all five pro-inflammatory indices are positively correlated with the DLR-score while LMR stands as the sole negatively correlated item. This symmetric “five-positive-one-negative” pattern is highly consistent with the immunological expectation that increased imaging heterogeneity accompanies aggravated peripheral pro-inflammatory imbalance and diminished anti-tumor immune surveillance, providing correlational evidence at the statistical association level supporting the hypothesis that ultrasound radiomics may noninvasively reflect systemic immune-inflammatory status.

To further examine whether BRAF V600E mutation status modulates the strength of imaging-immune coupling, Spearman correlations between DLR-score and NLR, SII, and LMR were computed separately for the BRAF-positive and wild-type subgroups and compared via Fisher z-transformation, as presented in **Table 3**.

**Table 3:** BRAF-stratified Spearman correlations between DLR-score and immune-inflammatory indices.

Index	BRAF+ ρ	BRAF− ρ	Fisher z	P value
NLR	0.399	0.156	3.777	< 0.001
SII	0.416	0.179	3.739	< 0.001
LMR	−0.281	−0.242	−0.595	0.552

Spearman ρ computed separately for BRAF-positive and wild-type subgroups. Fisher z-transformation tests the significance of the difference between two correlation coefficients.

**Table 3** shows that the BRAF-positive subgroup exhibits notably stronger DLR-score correlations with NLR and SII than the wild-type subgroup while the difference for LMR does not reach statistical significance, suggesting that BRAF mutation status may selectively strengthen the coupling between imaging heterogeneity and neutrophil-related pro-inflammatory signals.

The above unadjusted stratified correlations, while informative, remained susceptible to confounding by clinical variables that co-vary with both peripheral inflammatory levels and imaging heterogeneity. To evaluate whether the BRAF-dependent differences in imaging-immune coupling persisted after accounting for such confounders, partial Spearman rank correlations between DLR-score and the three representative indices were computed within each BRAF subgroup after adjusting for age, maximum tumor diameter, multifocality, and Hashimoto’s thyroiditis status, as presented in **Table 4**.

**Table 4:** Covariate-adjusted partial Spearman correlations between DLR-score and immune-inflammatory indices stratified by BRAF status.

Index	BRAF+ partial ρ	BRAF− partial ρ	Fisher z	P value
NLR	0.395	0.144	3.879	< 0.001
SII	0.412	0.167	3.824	< 0.001
LMR	−0.281	−0.229	−0.789	0.430

Partial Spearman ρ adjusted for age, maximum tumor diameter, multifocality, and Hashimoto’s thyroiditis. BRAF-positive n = 505, BRAF-negative n = 347. Bonferroni-corrected threshold for three comparisons: α = 0.017.

**Table 4** demonstrates that after adjustment for the four clinical covariates, the BRAF-positive subgroup retained substantially stronger partial correlations between DLR-score and both NLR with partial ρ = 0.395 and SII with partial ρ = 0.412 compared with partial ρ = 0.144 and 0.167 in the BRAF-negative subgroup, and the Fisher z-transformation confirmed that these between-group differences remained statistically significant after Bonferroni correction for three comparisons with z = 3.879 and P < 0.001 for NLR and z = 3.824 and P < 0.001 for SII. The attenuation from unadjusted to adjusted correlations in the BRAF-positive subgroup was minimal, with NLR decreasing from ρ = 0.399 to partial ρ = 0.395 and SII decreasing from ρ = 0.416 to partial ρ = 0.412, indicating that the four clinical covariates accounted for a negligible proportion of the original imaging-immune correlation and that the BRAF-stratified coupling pattern was largely independent of these confounders. The difference for LMR remained non-significant after adjustment with z = −0.789 and P = 0.430, consistent with the unadjusted analysis. These covariate-adjusted results strengthen the inference that the enhanced imaging-immune coupling observed in BRAF V600E-mutant PTC is not an artifact of confounding by tumor size, multifocality, age, or autoimmune comorbidity, but instead reflects a BRAF-associated biological modulatory effect that operates preferentially through neutrophil-related pro-inflammatory pathways.

The immunological plausibility of the above peripheral-imaging associations was further examined using publicly available tissue-level data as indirect corroboration rather than as external validation of the prediction model. If BRAF-positive PTC tissues exhibit elevated neutrophil infiltration and reduced CD8^+^ T cell proportions in an independent dataset, this would provide directionally consistent circumstantial support for the peripheral index patterns observed in the present cohort, as presented in **Figure 7**.

**Figure 7:**
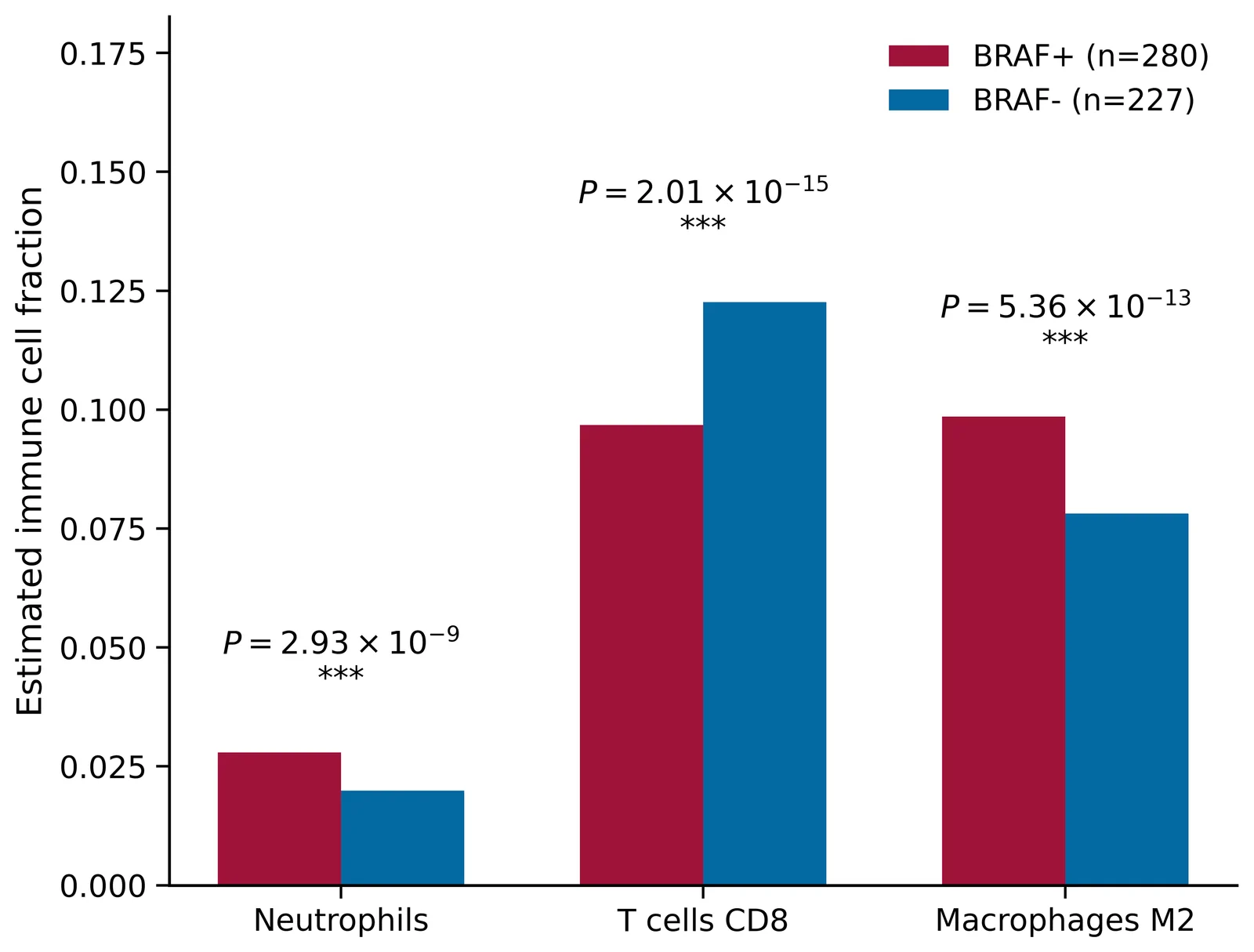
TCGA-THCA immune infiltration profiles stratified by BRAF status. Estimated immune cell fractions derived from CIBERSORTx (LM22 signature) for 507 PTC cases. P values from Mann-Whitney U tests. ***P < 0.001.

**Figure 7** shows that in the TCGA-THCA cohort, BRAF-positive PTC tissues exhibit higher neutrophil infiltration proportions than the BRAF-negative group, markedly lower CD8^+^ T cell proportions representing the most prominent difference among the three cell types, and higher M2 macrophage proportions. This tissue-level immune infiltration pattern is directionally consistent with the peripheral observations of elevated NLR and SII and decreased LMR in BRAF-positive patients in the present cohort, providing cross-level indirect corroboration from an independent public dataset and supporting the biological plausibility of the core hypothesis that peripheral immune-inflammatory signals may partially reflect the immune status of the tumor microenvironment.

### 3.5 Calibration and clinical utility

Beyond discriminative performance and biological plausibility, the clinical translation of a prediction model further requires accuracy of the output probabilities themselves and net benefit superiority over extreme strategies across clinically relevant decision thresholds. For the decision of whether to perform prophylactic central compartment dissection in cN0 PTC, clinicians typically weigh the benefits of dissection against surgical risks within a metastasis probability range of 10% to 50%, making the sustained provision of positive net benefit by the model within this threshold interval directly relevant to clinical practice, as presented in **Figure 8**.

**Figure 8:**
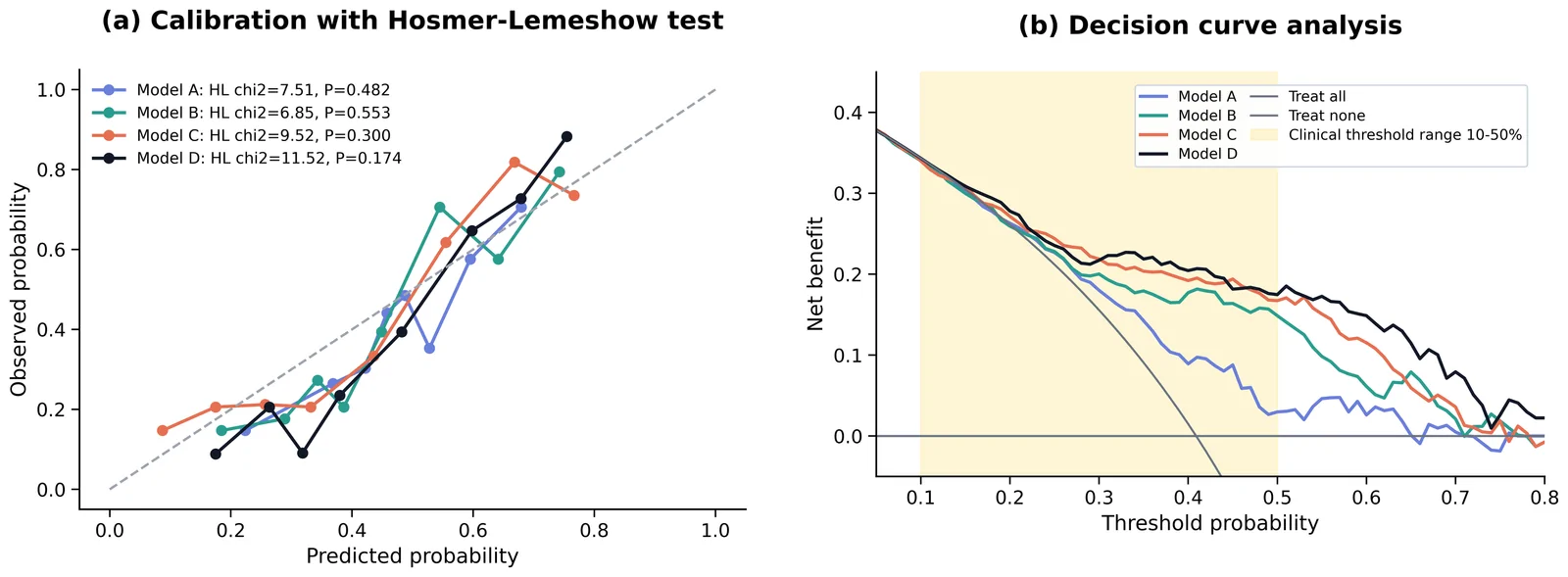
Calibration and clinical utility of the integrated model. **(a)** Calibration curves with Hosmer-Lemeshow test statistics. **(b)** Decision curve analysis with the clinically relevant 10% to 50% threshold range shaded.

According to (**Figure 8a**), it can be seen that the calibration graph demonstrates that Model D aligns well with the diagonal line in the middle range of probabilities. This suggests that there is a high level of agreement between the predicted probabilities and the incidence rates observed, meaning that besides its discriminative ability, the three-scale approach provides credible probabilities as well. As shown in (**Figure 8b**), Model D sustains greater net benefit relative to the other three models across all clinical threshold probabilities. It also outperforms the extreme “treat all” and “treat none” policies throughout, with more significant gains at low to moderate threshold probabilities. The above conclusion shows that the three-level integration model is able to help in avoiding unnecessary surgeries without losing the benefits of dissection. In addition, the individualized risk stratification tool proves to be practically beneficial to assist in making prophylactic central compartment dissections for cN0 PTC patients.

## 4 Discussion

### 4.1 Summary of key findings

This study constructed an integrated prediction model incorporating immune-related signals from three noninvasive scales of molecular mutation, systemic immune inflammation, and local imaging heterogeneity in a cohort of 1,000 cN0 PTC patients. The three-scale integration achieved the highest discriminative performance among all model configurations, with each scale contributing a statistically significant independent increment and the systemic immune scale providing the largest marginal gain. Beyond prediction, the cross-scale correlation analyses revealed a systematic directional alignment between imaging heterogeneity features and peripheral immune-inflammatory indices that was selectively strengthened in the BRAF-positive subgroup for neutrophil-related pathways. This pattern of selective enhancement persisted after adjustment for age, tumor size, multifocality, and Hashimoto’s thyroiditis in supplementary partial correlation analyses, suggesting that the observed BRAF-dependent modulation of imaging-immune coupling is not fully explained by confounding from these clinical covariates and may reflect a biological modulatory effect operating preferentially through neutrophil-related pro-inflammatory pathways. Tissue-level immune infiltration data from the TCGA cohort provided directionally consistent indirect support for these peripheral-imaging associations.

### 4.2 Biological plausibility and cross-scale corroboration

This imaging-immune cross-scale association received directional support from existing cross-cancer studies. In non-small cell lung cancer, radiomic features have been demonstrated to reflect the spatial heterogeneity of the tumor immune microenvironment (46). Research achieving noninvasive characterization of the tumor immune microenvironment through longitudinal CT radiomics has shown that quantitative imaging features can track dynamic changes in immune status (47). In the PTC field, multi-omics analysis has independently confirmed a significant association between BRAF mutation status and tumor immune infiltration patterns (48). Evidence from these different cancer types and omics levels provides directionally consistent mutual support with the cross-scale associations observed at the noninvasive level through peripheral blood immune indices and radiomic features in the present study.

### 4.3 Comparison with existing literature

In terms of predictive performance, existing studies have achieved clinically meaningful discriminative results in CLNM prediction through clinical-radiomic combined modeling (49), multimodal ultrasound fusion radiomics (50), multi-center multi-region radiomic approaches (51), and radiomic nomograms targeting papillary thyroid microcarcinoma subgroups (52). The AUC of the present model falls within the upper range of reported values, yet the primary contribution of this study lies not in incremental performance improvement but in the systematic exploration of cross-scale associations between radiomic patterns and immune-inflammatory parameters while maintaining competitive predictive accuracy.

### 4.4 Clinical implications and translation

The practical clinical translation of the proposed framework benefits from the fact that all three input signal sources, namely BRAF V600E testing, routine preoperative complete blood counts, and grayscale thyroid ultrasound imaging, are already standard components of the preoperative workup for cN0 PTC patients at most institutions, and model deployment therefore requires no additional examinations or costs. The decision curve analysis demonstrated that the integrated model provides sustained net benefit over both single-scale alternatives and extreme treat-all or treat-none strategies across the 10% to 50% threshold probability range within which clinicians most commonly weigh the benefits of prophylactic central compartment dissection against surgical morbidity. The probability calibration further confirmed that model-generated risk estimates closely approximate observed event rates, a prerequisite for patient-level counseling in which predicted probabilities directly inform shared decision-making. Taken together, these results suggest that the tri-scale framework could be operationalized as a preoperative risk stratification tool that identifies cN0 PTC patients at sufficiently elevated CLNM risk to warrant prophylactic dissection while sparing lower-risk patients from unnecessary surgical intervention, although prospective implementation studies evaluating workflow integration and clinical acceptance remain necessary before adoption.

### 4.5 Limitations and future directions

This study has several limitations. The single-center retrospective design inevitably introduces selection bias, and generalizability requires validation from multi-center cohorts. The ethics design as a non-tissue non-blood retrospective medical record study precluded obtaining tissue immunohistochemistry, flow cytometry, or single-cell sequencing data from the study cohort, rendering it impossible to directly quantify the correspondence between peripheral immune-inflammatory indices and tumor-infiltrating immune cell populations at the tissue level. The cross-scale associations reported in this study, including the Spearman correlations between DLR-score and peripheral immune-inflammatory indices, the BRAF-stratified moderation effects, and the TCGA tissue-level corroboration, are therefore exclusively correlational in nature and do not constitute mechanistic evidence that these noninvasive signals originate from a shared immunological process. Establishing such a link would require prospective cohort designs that simultaneously acquire multiplex immunohistochemistry or immunofluorescence data, flow cytometry-based immune cell quantification, and matched imaging and peripheral blood samples from the same patients, with single-cell RNA sequencing of tumor-infiltrating immune populations and spatially resolved transcriptomics potentially clarifying whether the imaging-derived heterogeneity features captured by the DLR-score correspond to specific immune cell spatial distribution patterns within the tumor microenvironment. Until such multi-modal functional evidence becomes available, the present findings should be interpreted as hypothesis-generating statistical associations that establish directional plausibility rather than definitive mechanistic equivalence across scales.

Peripheral immune-inflammatory indices are inherently susceptible to confounding by factors that were not systematically captured in this retrospective dataset. Although overt infections within two weeks before surgery and concurrent immunosuppressive medication use were excluded by the eligibility criteria, subclinical chronic inflammatory conditions such as periodontitis, low-grade metabolic inflammation associated with obesity or type 2 diabetes, and dyslipidemia-related endothelial activation may have independently elevated NLR or SII values irrespective of tumor biology. Stratification by concurrent Hashimoto’s thyroiditis was performed at the subgroup analysis level, yet the interaction between autoimmune thyroid inflammation and tumor-associated immune dysregulation could not be fully disentangled within the current design. The standardization of blood sampling to fasting morning collections within a seven-day preoperative window partially mitigated circadian and acute-phase variability, but inter-individual differences in chronic baseline inflammation remain an uncontrolled source of noise. Discriminative performance varied across five ultrasound device models, and gray-level normalization mitigated but did not fully eliminate device heterogeneity. BRAF testing did not cover all enrolled patients. The predictive models retained the full cohort through a missing indicator approach, whereas the BRAF-stratified cross-scale correlation analyses were necessarily restricted to the 852-patient sub-cohort with definitive mutation status. Although sensitivity analysis excluding BRAF confirmed directional consistency of the core conclusions, the representativeness of the tested sub-cohort for BRAF-stratified analyses warrants further verification.

An additional interpretive caveat concerns the immunological specificity of the peripheral blood indices employed in this study. NLR, PLR, LMR, SII, SIRI, and PIV are derived from routine complete blood count parameters and reflect the aggregate balance among circulating innate and adaptive immune cell populations at a systemic level. These indices cannot distinguish between tumor-directed immune responses and non-specific inflammatory processes unrelated to the thyroid malignancy, nor can they characterize antigen-specific CD8^+^ cytotoxic T cell activity, regulatory T cell suppression, or myeloid-derived suppressor cell expansion within the tumor microenvironment. The terminology “immune-inflammatory indices” adopted throughout this manuscript refers to their established nomenclature in the oncological literature and does not imply that these markers provide direct characterization of local tumor immunity. Although the TCGA-based tissue immune deconvolution analysis was incorporated to partially bridge this gap, its data originate from a different population and rely on computational estimation rather than direct cell counting, limiting its applicability to the present cohort. The development of noninvasive liquid biopsy approaches capable of quantifying tumor-derived immune signatures in peripheral blood, such as circulating tumor DNA methylation patterns reflecting immune checkpoint gene expression or extracellular vesicle-based immune cell profiling, may provide more immunologically specific systemic biomarkers for future iterations of the cross-scale analytical framework proposed here.

## 5 Conclusion

This study constructed a preoperative CLNM prediction model by integrating immune-related signals from three noninvasive scales, namely BRAF V600E mutation status, peripheral blood immune-inflammatory indices, and ultrasound radiomics score, based on a cohort of 1,000 cN0 PTC patients. The three-scale fully integrated model achieved a validation AUC of 0.824, with all scale-specific increments confirmed to be statistically significant by DeLong testing, among which the immune-inflammatory indices provided the largest single-step increment over the molecular baseline. Model interpretability analysis demonstrated that the interaction features between imaging and immune signals occupied a dominant role in prediction decisions, calibration assessment and decision curve analysis confirmed the probability accuracy and net benefit superiority of the model within clinically relevant threshold intervals, and auxiliary classifier comparison and sensitivity analysis excluding BRAF verified the robustness of the core conclusions.

Outside the model framework, directionally consistent cross-scale associations were observed between radiomic features and all six immune-inflammatory indices, with imaging-immune coupling significantly strengthened in the BRAF-positive subgroup while no significant difference was observed for the LMR dimension, and tissue-level immune infiltration data from the TCGA cohort provided directionally consistent indirect corroboration across key cell types. These findings provide hypothesis-generating statistical evidence suggesting that noninvasive immune signals captured at different biological scales may reflect partially overlapping aspects of tumor immune dysregulation, although the correlational nature of the present analyses precludes definitive mechanistic interpretation. Prospective studies integrating direct tissue-level immune profiling are warranted to validate the biological basis of these cross-scale associations. This analytical framework, shifting from a prediction-oriented to a relationship-oriented perspective, is expected to provide a methodological reference for exploring PTC metastasis from an immunological standpoint and to offer preliminary multi-scale evidence that may inform individualized preoperative decision-making for cN0 PTC patients.

## Data Availability

The original contributions presented in the study are included in the article. Further inquiries can be directed to the corresponding author.
